# Socioeconomic disadvantage and ethnicity are associated with large differences in children’s working memory ability: analysis of a prospective birth cohort study following 13,500 children

**DOI:** 10.1186/s40359-022-00773-0

**Published:** 2022-03-15

**Authors:** Kate E. Mooney, Kate E. Pickett, Katy Shire, Richard J. Allen, Amanda H. Waterman

**Affiliations:** 1 Department of Health Sciences, Seebohm Rowntree Building, University of York, York, YO10 5DD UK; 2 Bradford Institute for Health Research, Bradford, BD9 6RJ UK; 3 Centre for Applied Education Research, Wolfson Centre for Applied Health Research, Bradford, BD9 6RJ UK; 4 School of Psychology, University of Leeds, Leeds, LS2 9JT UK

**Keywords:** Working memory, Socioeconomic status, Ethnicity, Inequalities, Cognitive development

## Abstract

Working memory is a limited capacity system that stores and processes information over short time periods and is essential for learning new information. Some studies have investigated the associations between socioeconomic position and working memory, however none have examined this across potentially dissociable aspects of working memory. Further, there are very few studies about children’s working memory differences across and within different ethnic groups. Therefore, there is a need to understand the potential associations between socioeconomic position, ethnicity, and different aspects of children’s working memory. We investigated children’s working memory (*n* = 15,154) by socioeconomic group, using a latent class measure of family socioeconomic position, and then by ethnic group. To account for potential problems in applying socioeconomic measures across different ethnic groups, we then examined associations using an ethnic-specific socioeconomic measure within the ethnic majority group (White British) and the largest ethnic minority group (Pakistani). We found a strong association between socioeconomic group at birth and working memory at age 7–10 years, where the difference between the least and most deprived socioeconomic groups was equivalent to at least a 1-year age difference. We also found substantial differences in working memory between nine ethnic groups that varied by working memory task, where the difference between groups was equivalent to an age difference of between 6 and 24 months. Finally, we found evidence for a socioeconomic gradient in working memory for White British children, but this was considerably reduced in Pakistani children. These findings show the importance of separating out different ethnic groups when investigating associations between socioeconomic position and cognitive function, and that researchers need to be mindful when applying socioeconomic measures across ethnic groups. Where possible, ethnic-specific measures of socioeconomic position should be developed and applied for studies like these. Future research considering the possible mechanisms behind associations between ethnicity and working memory, and mechanisms by which socioeconomic position differentially influences working memory performance for different ethnic groups would shed further light on this important topic.

**Supplementary Information:**

The online version contains supplementary material available at 10.1186/s40359-022-00773-0.

## Background

Working memory is a limited capacity system that stores and processes information over short time periods to support ongoing cognitive activity [1, 2]. Working memory is essential for successful engagement in classroom activities [3], including the ability to remember and follow directions and instructions, and to engage effectively with learning and problem-solving [4–6]. In mathematics, working memory is required to hold solutions to intermediate steps whilst processing the remaining steps [7], and when reading working memory is required to keep relevant speech sounds in mind, match them with corresponding letters, and combine them to read words [8]. Working memory has strong associations with different learning outcomes, including mathematical performance [9] and broad reading abilities [10].

Socioeconomic position refers to the social and economic factors that determine what position an individual holds in society [11]. Several observational studies find socioeconomic position to be unrelated to children’s working memory [12–14]. However, other studies have found that socioeconomic disadvantage is associated with lower working memory ability [15–18]. There is inconsistency in this previous research investigating socioeconomic position and working memory, with some using tasks that rely primarily on storage (e.g. a forward’s spatial recall task) [18], some using tasks that require the processing of stored information (e.g. the Backwards Digit Recall (BDR) task) [16, 17], and others combining both types of task into a composite score [13]. This may, in part, account for the different findings relating to the association between socioeconomic position and working memory. Indeed, it has been found that children from low income groups have lower overall working memory scores compared to children from high income groups. However, low income children living in rural areas had disproportionately lower visuospatial (relative to verbal) working memory scores, whilst low income children living in urban areas had equal verbal and visuospatial working memory scores [19]. The association between socioeconomic position and working memory may therefore be biased when tasks measuring different aspects of working memory are combined into composite measures.

Influential modular theories of working memory propose distinct components within working memory including an executive control system for processing information, and two separable storage sub-systems for holding verbal and visuospatial information respectively [1]. These separate components may underpin the findings from Tine [19], where there were dissociable patterns of performance by modality, and may explain the differences in findings across studies that use tasks that require storage, processing, or a composite of both. It should be noted that other prominent theories see working memory as a more unified construct and do not support the idea of separable, specialized working memory components [2, 20]. However, despite debate around the precise nature of working memory, it is still important to gauge a child’s working memory profile across different input modalities, materials, and levels of task complexity, in line with natural variation in working memory task contexts encountered in the real world. Indeed, many standardised measures of working memory contain tasks that differ by modality presentation (e.g., using verbal versus visuospatial stimuli) or by whether information needs simply to be stored (e.g., forward digit span task) or to be manipulated in some way (e.g., reading span task). It is therefore important to explore whether the associations between working memory and socio-demographic factors differ by working memory task. Indeed, early work into socioeconomic disparities in executive functions found that the size of the socioeconomic difference was dependent on the type of executive function task [18, 21]. It is therefore possible that socioeconomic position will also have different effects on dissociable working memory tasks.

Another important issue to consider when investigating socioeconomic position and working memory is the measure of socioeconomic position that has been applied. Previously, studies regarding the association between socioeconomic position and working memory have typically applied standard socioeconomic measures (e.g., parental education, family income). However, previous research has found that response rates to income questions can be low and biased [22]; and this may result in a biased understanding of any associations between socioeconomic position and working memory. Instead, subjective measures (e.g. questions about how a family are coping financially) can capture aspects of socioeconomic disadvantage that standard measures do not [23, 24].

Ethnicity is an important factor to consider within the context of socioeconomic position and developmental outcomes, as minority ethnic groups tend to experience lower socioeconomic position [25]. Despite the potential importance of ethnicity to children’s working memory, few studies have investigated the differences in working memory between ethnic minority and majority children. The few published studies show that ethnic minority children tend to have lower working memory scores [17, 26, 27]. However, these studies either included very few different ethnic groups, or combined all ethnic minority scores into one heterogeneous group [26]. Related to this, another significant aspect of socioeconomic measurement is the degree to which it can be measured validly and reliably within ethnic minority groups. Research has contested the extent to which education accurately reflects socioeconomic position for ethnic minority groups, as this may depend on whether immigrants' qualifications are obtained in the country they are residing in [22].

A further issue is that none of these studies regarding ethnicity and working memory examined the associations between socioeconomic position and working memory *within* different ethnic groups*.* Recent research suggests the importance of stratifying by ethnic group to understand the true underlying associations between socioeconomic position and other outcomes. For example, in comparison to most other ethnic groups, White children at the lowest levels of socioeconomic position tend to be at higher risk for lower social emotional scores in the U.S. [28] and low educational achievement in England [29]. However, the reasons behind these disproportionately lower scores for White children are yet to be understood. Of course, the relationship between ethnicity and working memory is likely to be influenced by a variety of other factors beyond socioeconomic position, such as differences in culture, and experiences of prejudice and racism [30–32]. However, a detailed exploration of the relationship between ethnicity, socioeconomic position, and working memory is the first crucial step for understanding these associations, given the lack of research in this area.

Furthermore, the extent to which social gradients exist within different ethnic groups is important to understand. Only one study has explored social gradients across ethnic groups with working memory ability, finding that socioeconomic disadvantage was associated with lower working memory scores for African American children, whilst White children at different levels of socioeconomic position had more similar working memory ability to one another. The authors suggest that White children may be buffered from the negative effects of poverty by positive parenting [33]. However, research in other outcomes suggests the contrary, where social gradients are less pronounced in ethnic minority groups than ethnic majority groups for maternal and child health [23, 24], and social and emotional scores [28].

These contradicting findings about social gradients in ethnic minority groups may be due to inaccurate and unstable measurements of socioeconomic position in ethnic minority groups. To examine differences by ethnicity, it is important to adjust for socioeconomic position appropriately, however, the extent to which traditional measures of socioeconomic position are valid in different ethnic groups is contested (e.g., educational attainment). In response to this problem, ethnic-specific measures of socioeconomic position have been developed, which are more informative when looking at socioeconomic differences within ethnic groups [34]. However, no study has yet investigated the association between ethnic-specific measures of socioeconomic position and children’s working memory outcomes. The contradicting findings about social gradients in ethnic minority groups and the difficulties with measuring socioeconomic position within these groups highlights the need for research into socioeconomic differences in working memory across different ethnic groups, using appropriate measures of socioeconomic position. The findings can then feed into research investigating how best to model the relationship between sociodemographic factors and cognitive ability, and how best to measure socioeconomic position within different ethnic groups.

### Current study

Using data collected in a large-scale (*n* = 13,500) longitudinal birth cohort study in the UK, we investigated children’s working memory abilities, across potentially dissociable aspects of working memory, by socioeconomic factors and ethnicity. To address the need to understand social gradients within different ethnic groups, we investigated how socioeconomic disadvantage affects children using a measure of socioeconomic position across all ethnic groups, and ethnic-specific measures of socioeconomic position within the ethnic majority and the largest ethnic minority group. First, we analysed children’s working memory by age in years to provide a benchmark against which to compare the magnitude of any socioeconomic or ethnic differences. Second, we analysed differences in children’s working memory by socioeconomic group, using a latent class measure of family socioeconomic position at birth that incorporates subjective assessments of financial status. Third, we analysed the differences in children’s working memory across nine different ethnic groups. Finally, to account for potential problems in applying socioeconomic measures across different ethnic groups, we examined associations with working memory of an ethnic-specific socioeconomic measure within the two largest ethnic groups in the cohort (White British and Pakistani).

## Methods

### Setting and participants

The Born in Bradford (BiB) longitudinal cohort study recruited 12,453 pregnant mothers, with 13,776 pregnancies, between March 2007 and December 2010 [35]. Bradford is a city in Northern England with an ethnically diverse population. According to the UK’s Index of Multiple Deprivation, areas within Bradford span the top 10% least deprived to the top 10% most deprived neighbourhoods in the UK. Overall, Bradford is based in a region with very high levels of deprivation [36]. In early 2020, 27% of children in the North of England were living in poverty before housing costs and 33% after housing costs, compared to just 20% before housing costs and 30% after housing costs in the UK as a whole [37]. The largest proportion of Bradford’s population (63.9%) identify themselves as White British, and the city has the largest proportion in England of people of Pakistani ethnic origin (20.3%) [38].

As part of the BiB study, a set of cognitive tasks including three tasks of working memory were administered to children aged 7 to 10 years in 89 Bradford schools between 2016 and 2019. Schools with high numbers of BiB children were targeted, but all children in the relevant classes were tested, leading to 15,154 children completing ≥ 1 working memory task(s). Ethnicity data were collected for the larger sample, and the socioeconomic data were linked from the BiB baseline questionnaire (administered when BiB mothers were pregnant) for the BiB children only. Ethical approval was obtained from the NHS Health Research Authority’s Yorkshire and the Humber—Bradford Leeds Research Ethics Committee (reference: 16/YH/0062) on the 24th March 2016. For the school based cognitive measures an ‘opt out’ consent model was used, and all methods were carried out in accordance with relevant guidelines and regulations. Further details regarding this can be found in the study protocol and detailed methods described elsewhere [39, 40].

## Measurements

### Working memory

Specific details on the measurements of working memory can be found in Hill et al. [40], and the key details are briefly described here. Three widely used measures of working memory were administered via a tablet: Forward Digit Recall (FDR), Backwards Digit Recall (BDR), and Corsi [5, 41]. The FDR and Corsi tasks are tasks that primarily measure storage (verbal and visuospatial, respectively), and the BDR is a task that measures the processing of stored information. In FDR, children were presented with a sequence of digits (via headphones) and asked to recall the sequence in order. The tasks progressed from sequence length three to six, with four trials for each sequence length, with a total of 16 trials. BDR was similar to FDR, but children were asked to recall the digits in reverse order. As this task is more difficult than FDR, the sequence length started at two digits and increased to sequence length five, with a total of 16 trials. In the Corsi spatial task, children were presented with nine randomly arranged squares on the screen and had to recall spatial sequences in the order that they were highlighted. Sequence length increased from three to six squares, with again total of 16 trials. Responses in all tasks were made via touchscreen. Scores were recorded as percentage correct. These measurement methods had been previously trialled and used with children in primary schools in a number of studies (e.g. [42, 43]).

### Age

Child age was recorded in both years and months. We report working memory by child age in years in Table 1 and analyse working memory by child age in months in the regression analyses. Any children with age missing were dropped from all analyses (2.95% of the sample).

**Table 1:** List of socioeconomic groups and their characteristics (Fairley et al. [34])

Class	Description
Least socioeconomically deprived and most educated”	Women currently and previously employed Father non-manual employment Women and fathers highly educated Up to date with bills Mortgage Not subjectively poor Not receiving means tested benefits Not materially deprived
“Employed, not materially deprived”	Women currently employed Father manual and non-manual employment Women and father medium levels of education Up to date with bills Mortgage Not subjectively poor Not receiving means tested benefits Not materially deprived
“Employed, no access to money”	Women currently and previously employed Father manual and non-manual employment Women and father’s medium levels of education Moderate behind with bills Mortgage and private renting Moderate subjective poverty Moderate receipt of means tested benefits Materially deprived in particular can’t afford holidays, money to replace goods and savings
“Benefits and not materially deprived”	Women low current employment Father manual employment and self-employed Women and father’s low levels of education, father’s education high don’t know response Up to date with bills Owns house outright Not subjectively poor High receipt of means tested benefits Not materially deprived
“Most economically deprived”	Women low current employment Father manual employment and unemployed Women and father’s low levels of education, father’s education high don’t know response Behind with bills Private renting and social housing Subjectively poor Highest receipt of means tested benefits Materially deprived

### Gender

Child gender was recorded as either male or female for all children who completed the working memory task(s). Although we do not report working memory by child gender, we do report the total number of females and males who completed the tasks in Table 1.

### Ethnicity

Ethnicity information was provided by the schools and was coded into 9 categories: Pakistani, Bangladeshi, Indian, Black/Black British, White British, Mixed, Gypsy/Irish Traveller, Other White, and Other. As White British are the ethnic majority group in England, we conceptualised White British children as the ethnic majority group and children in all other ethnic groups as ethnic minorities. Whilst Pakistani children are the majority ethnic group in the Born in Bradford cohort (and in our sample), they are still an ethnic minority relative to White British children, in both Bradford and in England.

### Socioeconomic position

We chose to use the term socioeconomic position. We used two categorisations of socioeconomic position which were estimated using Latent Class Analysis (LCA) [34]. Fairley et al. [34] estimated socioeconomic position categories for the BiB cohort for examining socioeconomic differences *across* ethnic groups, and ethnic-specific categories of socioeconomic position for examining socioeconomic differences *within* two ethnic groups—and found slightly different classes when socioeconomic position was stratified by ethnic group (as described in the following section). In both cases, socioeconomic position categories were estimated using 19 variables relating to employment, education, benefits, and material deprivation collected during the baseline questionnaire (when the mother was pregnant with the child). In our analysis of the effect of socioeconomic position on working memory we used the first set of socioeconomic position classifications, which included “Least socioeconomically deprived and most educated”, “Employed and not materially deprived”, “Employed and no access to money”, “Benefits^1^ and not materially deprived”, and “Most economically deprived” [34]. These groupings combine many different dimensions of socioeconomic position into one overall measure, by grouping those with similar characteristics. The characteristics of the groups are provided below in Table 1, and further detail is available in the paper where the model was developed [34].

For our analyses of the effect of socioeconomic position on working memory within the two largest ethnic groups in the cohort—White British and Pakistani—we used the ethnic-specific classifications of socioeconomic position. The LCA stratified by ethnic group found that a 4-class model was the best fitting for both groups, however, the groups had different characteristics and therefore have different labels. Differences between ethnic groups were found in woman’s employment status and education, housing, subjective poverty, and material deprivation. The White British classes are: “Employed, educated, not materially deprived”, “Employed, moderate education, materially deprived”, “Low education, benefits, not materially deprived”, and “Low education, benefits, subjectively poor and materially deprived”. The Pakistani classes are: “Educated, low benefits, not materially deprived”, “Women employed, moderate education, benefits, not materially deprived”, “Women not employed, low education, benefits, not materially deprived”, and “Women not employed, moderate education, benefits, subjectively poor and materially deprived”. A key difference between the two sets of socioeconomic position categories is that within the White British group two classes were described as materially deprived, whereas only one class were materially deprived within the Pakistani group [34]. For a detailed description of the socioeconomic characteristics within each group alike Table 1, please see the paper where the model was developed [34].

We also report results for a simple measure of self-reported financial situation as this measure can be used on the same scale within both ethnic groups and has been shown to have good discriminatory power within this cohort [24]. Self-reported financial situation was assessed during the BiB baseline questionnaire, where participants were asked how well the family were coping financially. The responses include: 1 (living comfortably), 2 (doing alright), 3 (just about getting by), 4 (quite difficult), 5 (very difficult), and 6 (does not wish to answer). Participants who responded ‘6 (does not wish to answer) were not included in the figures.

### Sample characteristics

Table 2 provides a summary of the sample characteristics. A larger cohort of children completed working memory tasks and had linked education data containing age, gender, and ethnicity (n = 15,154), and a subset of these children were also enrolled in Born in Bradford (n = 5976).

**Table 2:** Socio-demographic characteristics of Bradford primary school children (*n* = 15,154) some of whom are also Born in Bradford cohort children (*n* = 5976)

Socio-demographic variable	Count	Percent
*Age (years)*		
7	5003	32.04
8	6726	43.08
9	3130	20.05
10	295	1.89
Missing	460	2.95
*Gender*		
Male	7480	49.36
Female	7674	50.64
Missing	0	0
*Ethnic group*		
Pakistani	6777	44.72
Bangladeshi	447	2.95
Indian	324	2.14
Black or Black British	264	1.74
White British	4137	27.30
Mixed	866	5.71
Gypsy or Irish traveller	168	1.11
White Other	677	4.47
Other	416	2.75
Missing	1078	7.11
*Socioeconomic group (BiB only, n* = *5976)*		
Least deprived and most educated	778	13.02
Employed, not materially deprived	843	14.11
Employed, no access to money	803	13.44
Benefits but coping	1659	27.76
Most deprived	833	13.94
Missing	1060	17.74

### Statistical analyses

Working memory outcomes are described by socioeconomic position and ethnicity using mean percentage correct scores and 95% confidence intervals. In addition to the mean percentage correct scores, we also present unstandardized regression coefficients and 95% confidence intervals for each of the independent variables (age, socioeconomic position, and ethnicity) on working memory. Regression coefficients were produced using simple linear regression in Stata-16 [44]. A statistically significant effect is not enough to inform us about the practical significance of an effect and depends heavily on a sample’s size [45], so we use regression coefficients and 95% confidence intervals as measures of effect size. The regression coefficients provide the predicted mean difference in percentage correct on each working memory task, between the baseline group and every other group.

First, we report the unstandardized coefficients and 95% confidence intervals for working memory by age differences in months. The coefficients from the age analysis are used as a benchmark for the regression coefficients in the socioeconomic position and ethnicity analysis—this allows a comparison of the magnitude of the effect between the socioeconomic and ethnic groups to differences in age. Next, we produced coefficients for working memory by socioeconomic position and ethnic group. The baseline group for socioeconomic position is the least deprived group, and the baseline group for ethnicity is the ethnic majority group (White British). Finally, responses by socioeconomic position are analysed within the two main ethnic groups (White British and Pakistani). We report mean working memory scores and 95% confidence intervals for all 3 working memory tasks across White British and Pakistani participants for two measures of socioeconomic position: (1) subjective financial status and (2) ethnic-specific socioeconomic position groups. We then produced the coefficients for working memory by the ethnic-specific measure of socioeconomic position, with the regressions stratified by ethnic group. We did not do this for subjective financial status as this was a variable included in the ethnic-specific measure, and the pattern of results appeared to be similar across both measures.

## Results

### Age

A summary table providing the unstandardized regression coefficients and 95% confidence intervals for age in months and working memory is provided in the supplementary online materials (see Additional file 1), and the key results are noted here. Overall, age in years was positively associated with all three tasks of WM. An age increase in 1 month was associated with the following: FDR (*β* = 0.36, 95% CI 0.33 to 0.39), Corsi (*β* = 0.55, 95% CI 0.51 to 0.58), and BDR (*β* = 0.57, 95% CI 0.54 to 0.61).

### Socioeconomic position

**Fig. 1:**
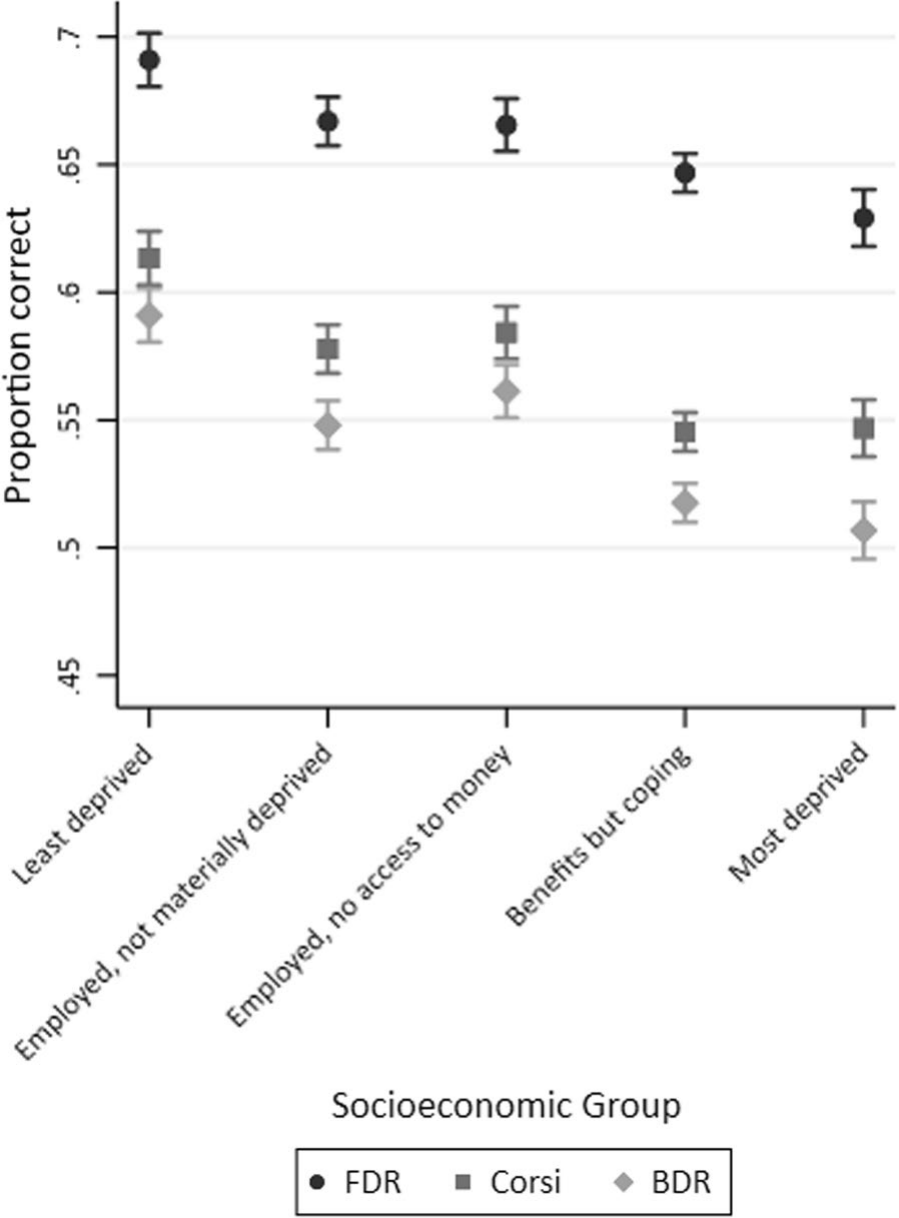
Mean scores and 95% confidence intervals in FDR, Corsi, and BDR by socioeconomic group (*n* = 4916)

Figure 1 shows that on average, the least deprived socioeconomic group had higher working memory scores than all other socioeconomic groups. Table 3 shows the linear regression results for each of the working memory tasks by socioeconomic group (where the reference group is least deprived).

**Table 3:** Regression results for FDR, Corsi, and BDR by socioeconomic group

Socioeconomic group	FDR (*n* = 4895)			Corsi (*n* = 4872)			BDR (*n* = 4913)		
	*B* (95% CI)	*t*	*p*	*B* (95% CI)	*t*	*p*	*B* (95% CI)	*t*	*p*
*Least deprived*									
Employed not materially deprived	− 2.47 [− 3.94 to − 0.99]	− 3.27	.001	− 3.52 [− 5.21 to − 1.84]	− 4.10	< .001	− 4.30 [− 6.13 to − 2.47]	− 4.60	< .001
Employed no access to money	− 2.28 [− 3.78 to − 0.78]	− 2.98	.003	− 2.70 [− 4.41 to − 0.99]	− 3.10	.002	− 2.97 [− 4.82 to − 1.11]	− 3.14	.002
Benefits but coping	− 4.23 [− 5.52 to − 2.94]	− 6.41	< .001	− 6.74 [− 8.22 to − 5.26]	− 8.96	< .001	− 7.34 [− 8.94 to − 5.73]	− 8.98	< .001
Most deprived	− 6.02 [− 7.51 to − 4.54]	− 7.97	< .001	− 6.56 [− 8.25 to − 4.86]	− 7.60	< .001	− 8.42 [− 10.26 to − 6.58]	− 8.98	< .001
F test	F(4, 4890) = 19.01			F(4, 4867) = 25.85			F(4, 4908) = 25.59		
Unadjusted R^2^, *p*	.02, < .001			.02, < .001			.02, < .001		

All groups scored significantly lower than the ‘least deprived’ socioeconomic group on all three tasks of WM. For the ‘employed, not materially deprived’ and ‘employed, no access to money’ groups, this difference was equivalent to a 5–7-month age difference for all three tasks. For the ‘benefits but coping’ group, this difference was equivalent to a 12–13-month age difference. For the ‘most deprived’ group, this difference was equivalent to a 12–18-month age difference. The size of the difference depended on the task, with FDR having smaller differences, and BDR having the largest differences.

### Ethnicity

**Fig. 2:**
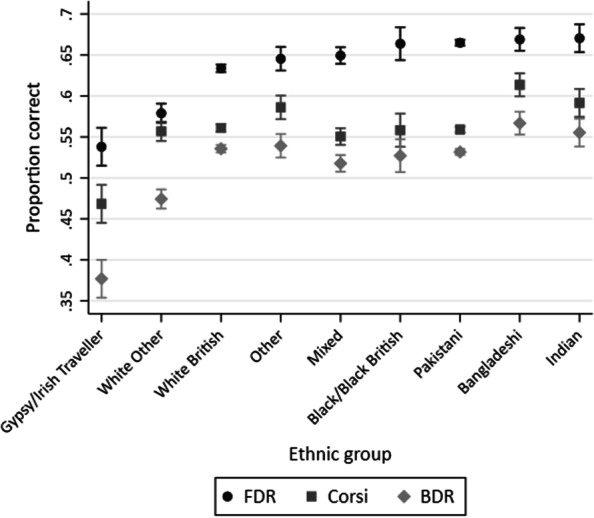
Mean scores in FDR, Corsi, and BDR by ethnic group ordered by FDR scores (*n* = 14,076)

Figure 2 shows the mean working memory scores for all 3 tasks of working memory for all ethnic groups, ordered by the mean score on FDR. Table 4 shows the linear regression results for each of the individual working memory tasks by ethnic group (when the reference is White British), with statistically significant results in bold.

**Table 4:** Regression results for FDR, Corsi, and BDR by ethnic group

Ethnic group	FDR (n = 14,025)			Corsi (n = 13,919)			BDR (n = 14,072)		
	*B* (95% CI)	t	*p*	*B* (95% CI)	t	*p*	*B* (95% CI)	t	*p*
*White British*									
Gypsy/Irish Traveller	− **9.58 [**− **11.93 to **− **7.23]**	− **7.98**	** < .001**	− **9.27 [**− **11.95 to **− **6.59]**	− **6.78**	** < .001**	− **15.87 [**− **18.82 to **− **12.92]**	− **10.56**	** < .001**
White other	− **5.48 [**− **6.72 to **− **4.24]**	− **8.66**	** < .001**	− 0.42 [− 1.83 to 1.00]	− 0.58	.564	− **6.14 [**− **7.69 to **− **4.58]**	− **7.75**	** < .001**
Other	1.16 [− 0.38 to 2.71]	1.47	.141	**2.51 [0.75 to 4.27]**	**2.79**	**.005**	0.36 [− 1.57 to 2.28]	0.36	.716
Mixed	**1.56 [0.44 to 2.68]**	**2.73**	**.006**	− 1.05 [− 2.33 to 0.23]	− 1.61	.108	− **1.79 [**− **3.19 to **− **0.39]**	− **2.50**	**.012**
Black/Black British	**3.00 [**− **2.45 to 1.90]**	**3.10**	**.002**	− 0.28 [− 2.45 to 1.90]	− 0.25	.802	− 0.85 [− 3.23 to 1.53]	− 0.70	.484
Pakistani	**3.12 [2.53 to 3.71]**	**10.36**	** < .001**	− 0.20 [− 0.87 to 0.47]	− 0.58	.382	− 0.39 [1.13 to 0.35]	− 1.03	.303
Bangladeshi	**3.53 [2.04 to 5.02]**	**4.64**	** < .001**	**5.26 [3.56 to 6.96]**	**6.06**	** < .001**	**3.12 [1.26 to 4.99]**	**3.28**	**.001**
Indian	**3.67 [1.94 to 5.40]**	**4.17**	** < .001**	**3.06 [1.09 to 5.02]**	**3.05**	**.002**	1.97 [− 0.19 to 4.13]	1.79	.073
F test	**F(8, 14,016) = 45.48**			F(8, 13,910) = 13.94			F(8, 14,063) = 24.55		
Unadjusted R^2^	**.03**, *p* **< .001**			**.00**, *p* **< .001**			**0.01**, *p* **< .001**		

Bold values shows the statistically significant results

Table 4 describes working memory scores by ethnic group. In comparison to White British children, Gypsy or Irish Traveller children had lower working memory scores in all three tasks, and this difference was equivalent to at least an 18-month age difference for Corsi, and a 2-year age difference for FDR and BDR. The ‘White Other’ group scored lower on two tasks (FDR and BDR), which was equivalent to approximately a 1-year age difference. The pattern for the other ethnic groups was mixed. All other children scored higher than the White British children for at least one of the working memory tasks. Pakistani and Black British children both had higher scores on the FDR task (equivalent to about a 9-month age difference), but not on the Corsi or the BDR tasks. In comparison to the White British group, the Bangladeshi and Indian children had higher working memory scores, and this different was equivalent to a 6–10-month age difference (dependent on the task).

### Associations between socioeconomic position and working memory within White British and Pakistani groups

**Fig. 3:**
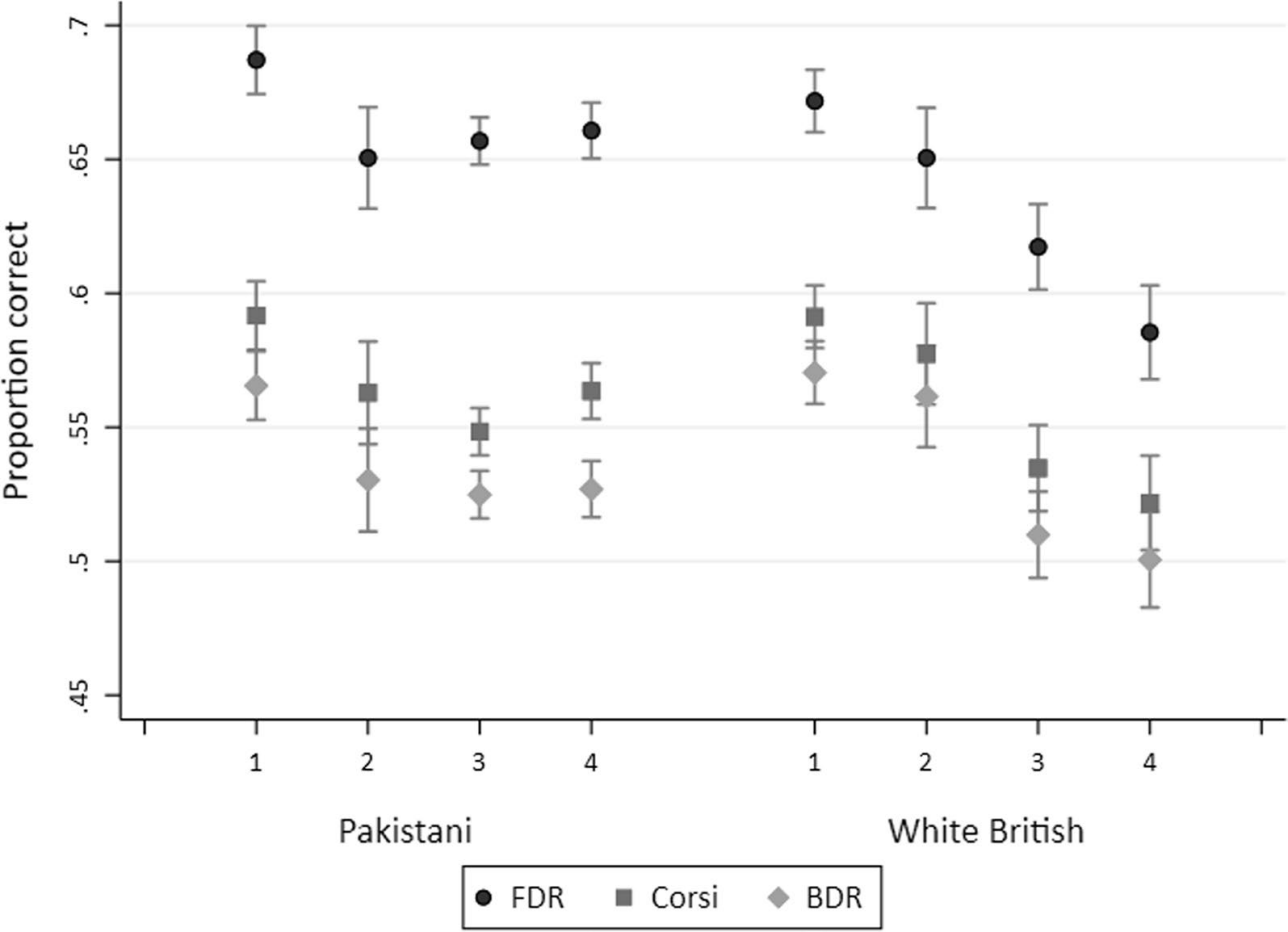
Mean FDR, Corsi, and BDR scores by ethnic specific latent class analysis of socioeconomic position for White British (*n* = 1517) and Pakistani (*n* = 2895) ethnic groups. Note: Pakistani classes included the following sample sizes: 1 “Educated, low benefits, not materially deprived” (n = 565), 2 “Woman employed, moderate education, benefits, not materially deprived” (n = 277), 3 “Woman not employed, low education, benefits, not materially deprived” (n = 1212), 4 “Woman not employed, moderate education, benefits, subjectively poor, materially deprived” (n = 841). White British classes included the following sample sizes: 1 “Employed, educated, not materially deprived” (n = 565), 2 “Employed, moderate education, materially deprived” (n = 275), 3 “Low education, benefits, not materially deprived” (n = 354), 4 “Low education, benefits, subjectively poor, materially deprived” (n = 323)

Figure 3 shows mean working memory scores across the ethnic-specific socioeconomic position measure in White British and Pakistani children. Socioeconomic position was a significant factor for White British children’s working memory, with those in the most deprived group having lower working memory scores than those in the least deprived group. For Pakistani children, socioeconomic position appears to have a weaker association with children’s WM, with those in the least deprived group only having slightly higher working memory scores than the other 3 groups. We also explored working memory by self-reported financial status (see Additional file 2), and the pattern of results was the same. White British children who were “living comfortably” had substantially higher working memory scores than the other groups, whilst Pakistani children who were “living comfortably” had similar working memory scores to those who reported “quite difficult” financial status. Tables 5 and 6 report regression results for the ethnic-specific socioeconomic position measure stratified by ethnic group.

**Table 5:** Regression results for FDR, Corsi and BDR by ethnic-specific socioeconomic position within White British children

Socioeconomic group	FDR (n = 1479			Corsi (n = 1469)			BDR (n = 1481)		
	*B* (95% CI)	t	p	*B* (95% CI)	t	p	*B* (95% CI)	t	p
Employed, educated, not materially deprived (baseline group)									
Employed, moderate education, materially deprived	− 1.82 (− 3.98 to 0.33)	− 1.66	.097	− 1.13 (− 3.56 to 1.29)	− 0.92	.358	− 0.89 (− 3.41 to 1.62)	− 0.70	.487
Low education, benefits, not materially deprived	− 5.09 (− 7.07 to − 3.11)	− 5.04	< .001	− 5.56 (− 7.78 to − 3.33)	− 4.90	< .001	− 6.05 (− 8.37 to − 3.73)	− 5.12	< .001
Low education, benefits, subjectively poor and materially deprived	− 7.99 (− 10.04 to − 5.94)	− 7.66	< .001	− 6.51 (− 8.81 to − 4.20)	− 5.55	< .001	− 6.98 (− 9.38 to − 4.59)	− 5.72	< .001
F test	F(3, 1475) = 22.36			F(3, 1465) = 14.57			F(3, 1477) = 16.08		
Unadjusted R^2^	.04			.03			.03

**Table 6:** Regression results for FDR, Corsi and BDR by ethnic-specific socioeconomic position within Pakistani children

Socioeconomic group	FDR (n = 2806)			Corsi (n = 2794)			BDR (n = 2818)		
	*B* (95% CI)	t	*p*	*B* (95% CI)	t	*p*	*B* (95% CI)	t	*p*
*Educated, low benefits, not materially deprived (baseline group)*									
Women employed, moderate education, benefits, not materially deprived	− 3.08 (− 5.32 to − 0.84)	− 2.70	.007	− 2.75 (− 5.34 to − 0.15)	− 2.07	.038	− 3.48 (− 6.37 to − 0.58)	− 2.35	.019
Women not employed, low education, benefits, not materially deprived	− 2.71 (− 4.26 to − 1.16)	− 3.42	< .001	− 4.21 (− 6.00 to − 2.42)	− 4.62	< .001	− 3.98 (− 5.98 to − 1.98)	− 3.91	< .001
Women not employed, moderate education, benefits, subjectively poor and materially deprived	− 2.43 (− 4.09 to − 0.78)	− 2.88	< .001	− 2.71 − 4.62 to − 0.80)	− 2.79	.01	− 3.73 (− 5.86 to − 1.60)	− 3.43	< .001
F test	F(3, 2802) = 4.56			F(3, 2790) = 7.12			F(3, 2814) = 5.62		
Unadjusted R^2^, *p*	.00			.01			.01		

Tables 5 and 6 report the regression results for working memory by the ethnic-specific socioeconomic position measure for White British and Pakistani children, respectively. For White British children, working memory scores decreased with each category of socioeconomic position (e.g., for FDR in reference to the least deprived group the differences were − 1.82, − 5.09, and − 7.99). For Pakistani children, each category of socioeconomic position was associated with lower working memory scores at a very similar magnitude (e.g., for FDR the differences were -3.08, − 2.71, and − 2.43). Further, the difference between the least and most deprived socioeconomic group for White British children was much larger, equivalent to a 12 to 18-month age difference, whilst the difference between the least and most deprived socioeconomic group for Pakistani children was equivalent to only a 6-month age difference.

## Discussion

### Socioeconomic differences in working memory

Our results consistently showed that socioeconomic disadvantage at birth was associated with lower working memory performance in middle childhood. This lends support to the view that socioeconomic position does influence working memory [16–19, 21, 46, 47], and contradicts the view that working memory is unrelated to socioeconomic disadvantage [12, 13]. In the current study the difference between the ‘most deprived’ and the ‘least deprived’ group was equivalent to a 12–18-month age difference. Previous literature on this topic was inconsistent in the type of task used to measure working memory, with some measuring storage, some measuring processing ability, and others combining these scores into a composite measure [13, 16–18].We were therefore interested to see if different patterns emerged when examining these working memory components separately. Our results showed that whilst the patterns were similar across working memory tasks, there were some differences in the magnitude of the effect. The largest gap was related to performance on the working memory task that measured the ability to process and manipulate information (the BDR task). This is in line with early research that suggested the size of the socioeconomic disparity depended on the type of cognitive task measured [18, 21], however, our study additionally suggests that socioeconomic disadvantage may have a more deleterious effect on the executive control aspect of working memory compared with simple storage. This also suggests that researchers should consider carefully whether it is appropriate to create composite scores of working memory when investigating these associations.

### Ethnic group differences in working memory

We found substantial variation in working memory scores by ethnicity, and this variation depended on the type of working memory task and on the ethnic group. Generally speaking, most ethnic minority groups scored higher than White British children on at least one measure of working memory This finding contrasts with the few previous studies looking at ethnic differences in children’s working memory, where it was generally found that ethnic minority groups tend to have lower working memory scores [17, 26, 27]. Since our study primarily focused on socioeconomic and ethnic group differences in working memory, the mechanisms behind ethnic differences in children’s working memory remain unexplained. This is an important area for future research, and we expand upon some possible mechanisms below.

For Bangladeshi and Indian children, this difference was equivalent to an age advantage of 6–10 months, depending on the task. For Black British and Pakistani children, this advantage was equivalent to an age difference of 9 months, but was only present for the FDR task, which measures the ability to store verbal information. Children from several of the ethnic minority groups in Bradford attend Islamic education at a Mosque or Madrassa, which often involves learning the Qu’ran verbally by rote. Memorisation techniques based on repetition have previously been suggested as an explanation for higher scores on FDR [48]. Whilst we do not have sufficient data to explore working memory by Mosque attendance for all ethnic groups, most Pakistani children in our sample (86%) did report attending Mosque or Madrassa, and these children had higher FDR scores than those who did not attend Mosque, where the difference was equivalent to 5-months (and was smaller for the other working memory tasks (≤ 2 months)). Therefore, learning the Qu’ran by rote may lead to an improved ability to store and repeat verbal information, which could impact performance on the FDR task. However, further research exploring these associations in more detail is necessary before any conclusions can be made.

Gypsy and Irish traveller children scored significantly below White British children, comparable to an age gap of at least 18 months. National data sources report that nearly a quarter of Roma, Gypsy, and Traveller children experience multiple forms of deprivation [49]. These children also have the lowest educational attainment in the UK, and experience high levels of bullying, racism, poor school attendance, and school exclusion [50, 51]. Practitioners describe how Roma, Gypsy and Traveller families often value different skills and knowledge that benefit from a more ‘holistic’ way of learning, including inclusion in real life projects that involve them as part of the community (e.g. farm work) [52]. These factors are likely to contribute to the differences in working memory between the Roma, Gypsy and Traveller children and children from the settled community.

### Socioeconomic position and working memory within White British and Pakistani groups

We found that self-reported financial status and an ethnic-specific measure of socioeconomic position were both associated with working memory for White British children, with those from lower socioeconomic position groups having lower working memory scores than the other groups. However, neither of these measures showed as strong an association with children’s working memory among Pakistani children.

Our findings contradict previous research where socioeconomic disadvantage was associated with lower working memory scores for ethnic minority children, but not ethnic majority children [33]. However, our results are consistent with previous research looking at health outcomes (e.g. preterm birth), which have also shown a lack of social gradients for Pakistani participants [23, 24]. Our findings are also consistent with research in other areas, where socioeconomically deprived White children are found to be most at risk for different outcomes (educational attainment and socio-emotional scores) [28, 29]. This may reflect that the measurement of socioeconomic position in ethnic minority groups is biased, and is not accurately detecting differences in socioeconomic position. However, we did use an ethnic-specific measure of socioeconomic position – which are a more accurate and reliable measure for the Pakistani ethnic group [34]. Alternatively, this may reflect that the detrimental effects of socioeconomic disadvantage for Pakistani children within low socioeconomic position groups are buffered by other factors, such as the high ethnic density of the Pakistani population within Bradford, and their strong social networks [53, 54].

### Strengths and limitations

The population in this study was drawn from one city within the UK, and reflects the ethnic groups living within that city. However, most of the major cities in the UK have areas where there are high levels of deprivation, and many have a population that contains several different ethnic groups. The results presented here are therefore generalizable beyond Bradford.

A limitation is that socioeconomic data and working memory measures were only available at one time point, however, the association between socioeconomic position at birth and later working memory ability is useful in suggesting the importance of the early years’ environment for longer-term cognitive development. Related to this, socioeconomic data was not available for all ethnic minority groups, and this means we cannot be certain how much of the ethnic differences in working memory scores would be explained by socioeconomic position. Nonetheless, this is the first study to provide an overview of differences in working memory by nine different ethnic groups.

Finally, this is one of the largest contemporary studies of working memory in children. A key strength is that we presented data looking at social gradients *within* the ethnic majority group and the largest ethnic minority group, using standard measures of socioeconomic position as well as ethnic-specific measures of socioeconomic position—which may be more appropriate and more valid for ethnic minority groups.

### Future research

A priority for future research is to establish the strength of these associations across the lifecourse. This can be achieved in studies such as BiB through multiple measurements throughout childhood, adolescence, and adulthood. Indeed, BiB is beginning a third phase of data collection called ‘Age of Wonder’, which will allow investigations into any changes in socioeconomic position over time, and how these relate to changes in working memory. Future research should also aim to establish the mechanisms behind the ethnic group differences in children’s working memory. It was not possible in our study to establish how much variance in ethnic group differences is due to higher or lower socioeconomic differences due to a lack of socioeconomic data in the smaller ethnic minority groups. However, studies should aim to establish this in other samples using mediation analyses. It is also important to establish the extent to which working memory scores are dependent on the situation and the culture they are embedded in, as many executive function tasks do not resemble ‘real-world’ activities, and this may bias the approximation of skills for children who are less familiar with such tasks. For a wider discussion about culturally responsive perspectives on ethnic minority children’s executive function abilities, we refer the reader to Miller-Cotto, Smith, Wang, and Ribner [30].

With regards to ethnicity, it is interesting to consider how the ethnic group differences in working memory map onto ethnic group differences in national educational attainment, where White children at low levels of socioeconomic position tend to have disproportionately lower attainment [29]. A question for future research may be whether low working memory scores for low socioeconomic position White British children can explain their lower-than-expected attainment in the national pupil database.

## Conclusion

This study found large socioeconomic and ethnic group differences in children’s working memory, and showed these factors interacted in their association with the development of this essential cognitive ability. Children from socioeconomically disadvantaged families scored less well on each of the working memory tasks compared to those children from the least deprived socioeconomic group, with the biggest gap reflecting difficulties with executive control abilities in the processing of information. Differences between ethnic groups revealed substantial difficulties for some ethnic minority children, although several ethnic minority groups showed better working memory ability than the majority White British group on at least one of the working memory tasks. Further, social gradients were evident within the ethnic majority group, but less so in the largest ethnic minority group. This may reflect important differences in how socioeconomic disadvantage interacts with ethnicity in influencing cognitive development.

## Supplementary Information

**Additional file 1**. Working memory scores by age (regression analysis).

**Additional file 2**. Working memory scores by subjective financial status within White British and Pakistani groups.

## Data Availability

Data can be made available upon request from Born in Bradford (see https://borninbradford.nhs.uk/research/how-to-access-data/). Code for data cleaning and analysis can be made available and be uploaded an open source repository once the paper has been conditionally accepted.

## Footnotes

1 ‘Benefits’ refers to any of six income supports received in the UK (since replaced by Universal Credit), and is alike to the US welfare system.
