# Supplementary material for: Socioeconomic disadvantage and ethnicity are associated with large differences in children’s working memory ability: analysis of a prospective birth cohort study following 13,500 children
Source: BMC Psychol. 2022 Mar 15;10:67. doi: 10.1186/s40359-022-00773-0 (PMC8925097; doi:10.1186/s40359-022-00773-0)
Supplement: Supplementary file 1 — Additional file 1. Working memory scores by age (regression analysis). [file 40359_2022_773_MOESM1_ESM.docx]

## Additional File 1: Age and working memory analysis

|  | **FDR (n = 15,087)** | | | **Corsi (n = 14,995)** | | | **BDR (n = 15,146)** | | |
| --- | --- | --- | --- | --- | --- | --- | --- | --- | --- |
|  | *B* (95% CI) | *t* | *p* | *B* (95% CI) | *t* | *p* | *B* (95% CI) | *t* | *p* |
|  | 0.36 (.33 to .39) | 24.36 | <0.001 | 0.55 (0.51 to 0.58) | 33.58 | <0.001 | 0.57 (0.54 to 0.61) | 31.79 | <0.001 |
| **F test** | ***F(1, 15085) = 593.39*** | | | ***F(1, 14993) = 1127.32*** | | | ***F(1, 15144) = 1010.73*** | | |
| **Unadjusted R^2^, *p*** | ***.04, p < 0.001*** | | | ***.07, p < 0.001*** | | | ***.06, p < 0.001*** | | |
