# Supplementary material for: Socioeconomic disadvantage and ethnicity are associated with large differences in children’s working memory ability: analysis of a prospective birth cohort study following 13,500 children
Source: BMC Psychol. 2022 Mar 15;10:67. doi: 10.1186/s40359-022-00773-0 (PMC8925097; doi:10.1186/s40359-022-00773-0)
Supplement: Supplementary file 2 — Additional file 2. Working memory scores by subjective financial status within White British and Pakistani groups. [file 40359_2022_773_MOESM2_ESM.docx]

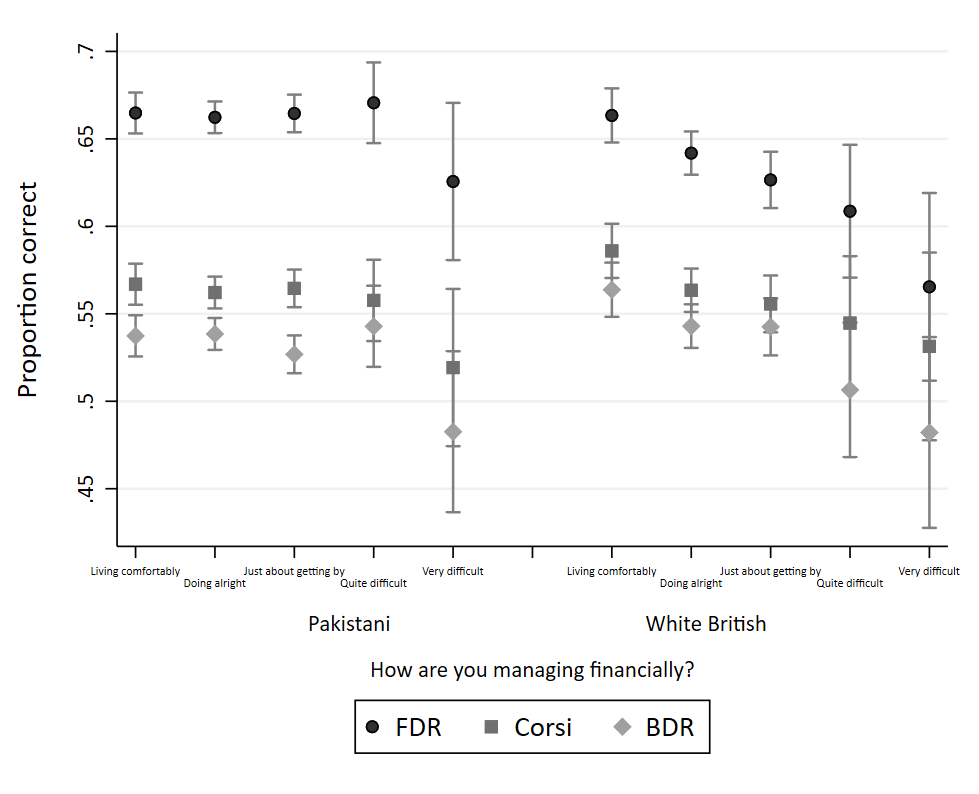
Additional File 2: WM and subjective financial status within White British and Pakistani groups

**Figure 4.** Mean WM scores by subjective financial status in Pakistani group and White British group
